# Use of a Piezosurgery Technique to Remove a Deeply Impacted Supernumerary Tooth in the Anterior Maxilla

**DOI:** 10.1155/2015/974169

**Published:** 2015-12-08

**Authors:** Shintaro Sukegawa, Takahiro Kanno, Kiyokazu Kawakami, Akane Shibata, Yuka Takahashi, Yoshihiko Furuki

**Affiliations:** ^1^Division of Oral and Maxillofacial Surgery, Kagawa Prefectural Central Hospital, No. 1-2-1, Asahi-cho, Takamatsu, Kagawa 760-8557, Japan; ^2^Department of Oral and Maxillofacial Surgery, Shimane University Faculty of Medicine, No. 89-1, Enya-cho, Izumo, Shimane 693-8501, Japan; ^3^Kawakami Orthodontic Clinic, No. 1-3-30, Ban-cho, Takamatsu, Kagawa 760-0017, Japan

## Abstract

Deeply impacted supernumerary teeth in the anterior maxillary cannot be generally removed by the conventional labial or palatal surgical approach because of the risk of damaging the surrounding soft tissues and the possibility of injuring the roots of adjacent permanent teeth. In piezosurgery, bony tissues are selectively cut, thereby avoiding the soft tissue damage caused by rotary cutting instruments. We report the case of a 15-year-old Japanese boy from whom a deeply impacted supernumerary tooth in the anterior maxillary was safely removed through the floor of the nasal cavity. The surgical extraction was performed without damaging the nasal mucosa or adjacent structures such as the roots of the adjacent permanent teeth. Considering that piezosurgery limits the extent of surgical invasion, this technique can be practiced as a minimally invasive and safe surgical procedure for treating suitably selected cases with a deeply impacted supernumerary tooth.

## 1. Introduction

Supernumerary teeth have been defined as teeth in excess of the usual human configuration of 20 deciduous and 32 permanent teeth [1]. The most common site of occurrence is the maxillary incisor region, with oral and maxillofacial surgeons sometimes encountering supernumerary teeth. These teeth are more prevalent among males than females, with a ratio of 2 : 1. The prevalence of supernumerary teeth is reported to be 0.3%–0.8% with deciduous dentition and 1.5%–3.5% with permanent dentition [2, 3]. Complications associated with supernumerary teeth include impaction, delayed eruption, ectopic eruption, spacing anomalies, and follicular cyst formation [4, 5]. However, excessive, deeply impacted supernumerary teeth that occur in the anterior maxilla cannot be removed because of the possibility of damaging the surrounding soft tissues and injuring the roots of adjacent teeth using a conventional labial or palatal surgical approach.

Piezosurgery utilizes amplified ultrasonic microvibrations as a minute cutting edge to cut bony tissue. This device has proven to be an effective tool for performing bone surgery procedures and makes the cutting of hard tissue possible through “selective cutting” without encountering necrosis from overheating as a result of friction and without damaging soft tissues [6, 7]. Although the advantages of ultrasonic surgery such as incomparable atraumaticity and precision in surgery are widely accepted by oral and craniomaxillofacial surgeons, its use for the safe removal of supernumerary teeth from the nasal cavity area has not yet been well documented.

In this report, we present the use of a piezosurgery technique for the safe removal of a deeply impacted supernumerary tooth in the anterior maxilla accessed through the floor of the nasal cavity.

## 2. Case Report

**Figure 1:**
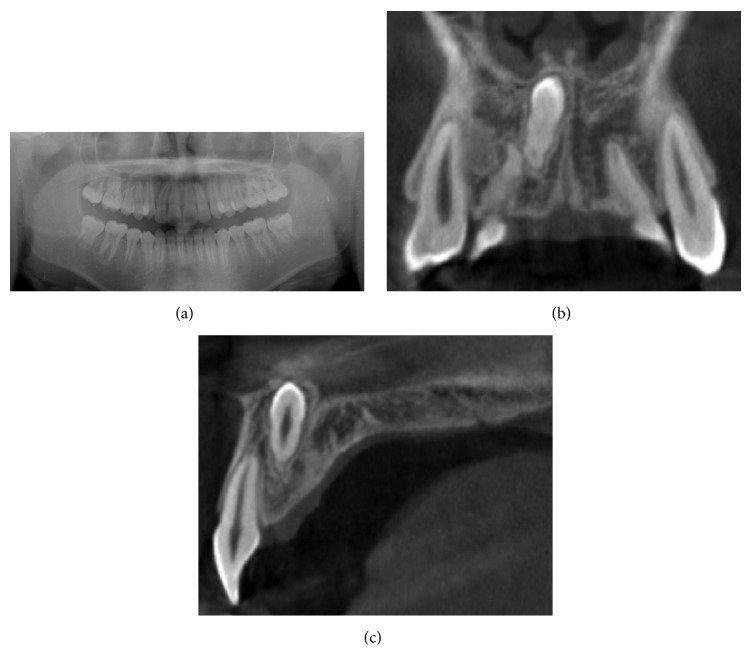
(a) Orthopantomograph X-ray and (b) coronal and (c) sagittal computed tomography images showing a deeply impacted supernumerary tooth in the anterior maxilla. The crown of the tooth was exposed on the nasal cavity side.

**Figure 2:**
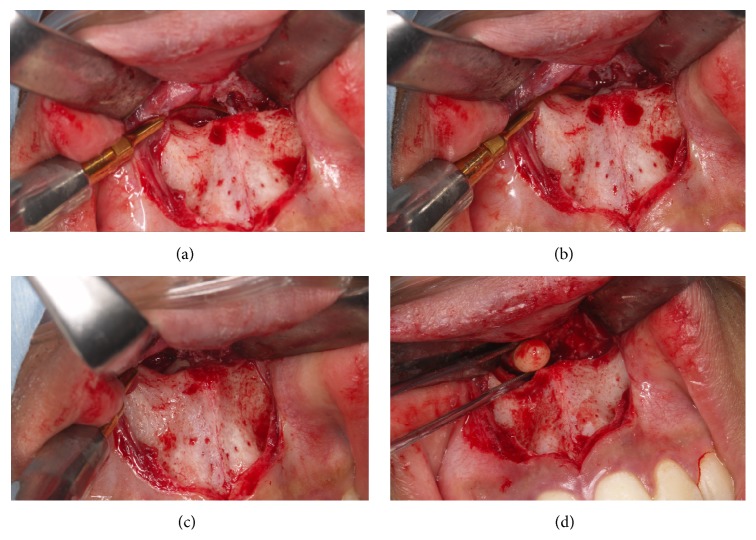
A trough around the crown of the impacted supernumerary tooth aided the use of piezosurgery from the floor of the nasal cavity in the restricted field of view. The buccal (a) and palatal (b) bone over the impacted supernumerary tooth (c) showing the uncovered impacted supernumerary tooth crown. (d) Extraction of deeply impacted supernumerary tooth of the anterior maxilla from the nasal cavity side.

In February 2015, a 15-year-old Japanese boy was referred to our hospital with a chief complaint of a deeply impacted supernumerary tooth. The patient had a history of orthodontic treatment at a local dental clinic. Intraoral examination showed normal dentition and no abnormalities. A panoramic radiograph showed 28 permanent teeth and a supernumerary intranasal tooth. Subsequent computed tomography (CT) showed that the crown of the supernumerary tooth was exposed to the nasal cavity side (Figure 1). Because tooth extraction using a conventional labial or palatal approach would not only be very challenging and invasive but also result in excessive bone removal, the patient instead underwent tooth extraction through the floor of the nasal cavity under local anesthesia and intravenous sedation. After making a vestibular incision from the lateral incisor to lateral incisor to create a “v” shape around the upper lip frenulum, a mucoperiosteal flap was raised from the labial aspect of the maxillary anterior teeth toward the piriform. The piriform rim was located, and subperiosteal dissection of the nasal mucosa was performed to expose the impacted supernumerary tooth. Using a piezosurgery device, a trough was created around the crown of the impacted supernumerary tooth from the floor of the nasal cavity, and the tooth was elevated and extracted (Figure 2). Careful attention was paid to avoid trauma and damage to the nasal mucosa and adjacent structures such as the roots of the adjacent permanent teeth and the nasopalatine nerve. The patient experienced no postoperative complications such as nose bleeding or damage to the adjacent teeth.

## 3. Discussion

**Figure 3:**
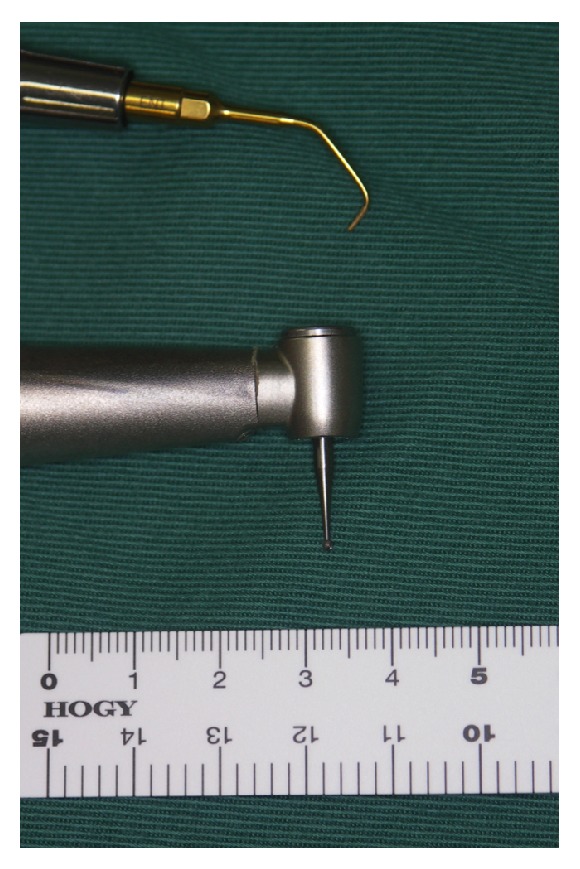
The head of the piezosurgery device is smaller than that of a rotary cutting instrument.

Supernumerary teeth are developmental alterations that may manifest in conjunction with both primary dentition and permanent dentition, can occur in both the maxilla and mandible, and can involve any tooth. In pediatric patients, a mesiodens is the most commonly impacted tooth [8]. When a supernumerary tooth causes symptoms or clinical signs, it becomes necessary to remove the tooth. In the current case, the crown of the impacted supernumerary tooth was located in the floor of the nasal cavity. This type of impacted supernumerary tooth is commonly removed using a labial or palatal approach when it is located near the alveolar process. However, conventional labial or palatal approaches for the removal of a deeply impacted supernumerary tooth require excessive bone removal and can potentially cause damage to adjacent structures such as the roots of the adjacent permanent teeth as well as potential nasopalatine nerve injury. Therefore, it was decided to remove the tooth from above (i.e., the nasal cavity side). For this procedure, it is desirable to reliably protect the nasal mucosa and to remove the smallest possible amount of bone. Traditional rotating burs are highly effective in cutting bone tissue, but they are not selective for bone and thus can cause significant harm to the surrounding soft tissues. In addition, because the head of a rotary cutting instrument is relatively large (Figure 3), it is challenging to maneuver in a narrow site. We instead performed the surgery using a piezosurgery device as a minimally invasive and safe approach for removing the deeply impacted supernumerary teeth in the anterior maxilla.

A piezosurgery unit is approximately three times as powerful as a conventional ultrasonic dental unit, allowing it to cut through highly mineralized cortical bone. The vibrations obtained are amplified and transferred to a vibration tip which, when applied with slight pressure on bone tissue, results in a cavitation phenomenon, producing a mechanical cutting effect that occurs exclusively on mineralized tissue [9]. In contrast to conventional rotary cutting instruments, to which the surgeon must apply a certain degree of pressure, the piezosurgery device needs only minimal pressure, permitting a more precise cut [10]. Various types of tips are available depending on the specific application and surgical site, including a scalpel, sharp-tipped saw, and several types of curved and/or straight tips. Selecting a tip according to the specific positioning and bone shape can ease a challenging approach toward the surgical site. In addition, with this case, an inverted, impacted tooth was located in the bottom of the nasal cavity and behind the central incisor. In this situation, selective bone cutting must be reliably and precisely performed with a narrow field of view to protect the nasal mucosa and to avoid damaging the root of the central incisor. The piezosurgery device proved to be extremely effective in this application.

Although the piezosurgery approach was a useful method for this case, the positional relationship between a deeply impacted supernumerary tooth in the anterior maxilla and all adjacent structures should be assessed preoperatively by CT, including three-dimensional images, when determining the optimal surgical approach.

## 4. Conclusion

Herein, we reported the use of the piezosurgery technique to remove a deeply impacted supernumerary tooth in the anterior maxilla. This approach can be utilized as a minimally invasive and safe surgical procedure for the removal of a suitably selected, deeply impacted supernumerary tooth.
